# Quality of life in children and adolescents with Osteogenesis Imperfecta: a qualitative interview based study

**DOI:** 10.1186/1477-7525-12-54

**Published:** 2014-04-16

**Authors:** Claire L Hill, Wendy O Baird, Stephen J Walters

**Affiliations:** 1 Metabolic Bone Disease Team, Sheffield Children's NHS Foundation Trust, Western Bank, Sheffield S10 2TH, UK; 2 School of Health and Related Research, University of Sheffield, Regent Court, 30 Regent Street, Sheffield S1 4DA, UK

**Keywords:** Children, Health and well-being, Osteogenesis imperfecta, Quality of life

## Abstract

**Background:**

Osteogenesis Imperfecta (OI) is a disease with varying severity affecting physical, social and emotional well-being of the child and their family. There is no existing evidence on how the OI population regard their quality of life (QoL). The main aim of this study was to determine how OI impacts on the quality of life and well-being of children and their family. It is the first stage of a larger project to develop a disease specific quality of life measure for children with OI.

**Methods:**

Purposive sampling was used to cover the diversity of the OI population. Twenty-five qualitative interviews were undertaken with children (n = 10), parents (n = 10) and health professionals (n = 5). Interviews were digitally recorded and transcribed verbatim. Significant themes were identified, extracted and organised, undergoing framework analysis.

**Results:**

Six main themes were identified; being safe and careful, reduced function, pain, fear, isolation, independence. There was a large amount of agreement between the three groups of interviewees, although discrepancies did occur between parents and children, with regard to the themes independence and fear.

**Conclusions:**

This data presents the first step in developing items for a disease specific QoL measure for children with OI. Several of the themes uncovered showed similarity to other QoL measures, but the addition of being safe and careful, particularly in relation to fractures, demonstrated the need for a disease specific measure for children with OI.

## Background

Osteogenesis Imperfecta (OI) is a hereditary condition affecting approximately 1 in 20,000 births with eleven recognized types of OI [1,2]. Children with OI have low bone mass, leading to recurrent fractures, varying degrees of short stature and deformity. Severity ranges from those mildly affected individuals (Type 1) who have minimal bone deformity, near normal stature, blue sclerae and variable hearing loss, to those who are lethally affected with multiple fractures in utero and respiratory failure [2].

Treatment regimes for children with OI aim to; provide pain relief; reduce fractures; prevent deformity, improve mobility and facilitate independent function. The current treatment of choice for children with moderate to severe OI is bisphosphonate therapy. Children treated with bisphosphonate therapy have reportedly shown an increased bone mineral density and reduction in fracture rate [3-6]. Anecdotal evidence that bisphosphonate treatment increased muscle strength, improved motor function and reduced pain are not supported by controlled trials [7]. Bisphosphonates decrease the efficiency of osteoclasts, the cells which break down bone, and therefore reduce bone resorption [8]. This results in thicker cortices; hence reduced long bone fractures, increased bone mineral density and reduce pain [9]. Thus, OI and its associated treatments can have a large impact on the health, well-being and quality of life (QoL) of the child and their family.

Previous attempts, at measuring QoL in OI, have used several generic instruments including; PEDI, WeeFim, Visual analogue scale, Bleck score, Health Utilities Index III (HUI III), and Self-perception profile for children (SPPC)[10-12] Although Seikaly et al. [10], using a visual analogue scale and number of pain free days, found significant improvement in wellbeing and reduction in pain between their two treatment groups they failed to identify any improvement in quality of life or function. Generic QoL measures such as the HUI III and the SPPC can be used to assess and compare a range of different disease states and healthy individuals but they may not be responsive enough to detect the small changes in QoL experienced by a child, with a particular disease or condition following treatment and clinically important aspects of the child’s life related to a specific disease may be overlooked [13].

Quality of life (QoL) is a multidimensional concept, which is individual and time dependent, incorporating physical, emotional and social aspects [14,15]. There is apparent discrepancy across the literature as to what dimensions should be included within a QoL measure, but most agree that it is a subjective measure and should therefore be evaluated by asking the patient.

Disease specific measures may be more sensitive to detect changes in QoL and are useful in comparing different interventions [16] or detecting treatment effects [17], but do not allow comparison with children with different diseases or conditions [15]. We could identify no disease specific QoL measure for children with OI.

Development of quality of life measures is often achieved from expert opinion, literature reviews and interviews with the relevant population [18]. Items are then developed and psychometrically tested on that population. The varied content and different dimensions within instruments is likely due to the developmental process, target population and proposed use [19].

Previously children have been thought to be unreliable respondents of quality of life measures and parents have been asked to provide this information [20]. Parent’s views are often affected by their own experiences of health, their knowledge, life experience and expectations [21]. However, studies suggest the views of children are required when developing new generic and disease specific paediatric quality of life measures to demonstrate good psychometric properties [15,19,22]. Children have demonstrated that they do not need their views represented by adults, they are capable of giving insights into their health from 5 years of age [23] and have been used to develop items and a descriptive system for preference based measures [18].

Previous studies [10-12] within the OI population have not attempted to understand children’s views on their health and quality of life. It is hypothesised that the development of a disease specific QoL measure for children with OI would improve outcome measurement following medical, surgical and therapy intervention. In order to create a disease specific QoL measure for OI children it is necessary to understand how OI impacts on the health and wellbeing of children and their family, and what issues are important to them.

The aim of this paper is to use qualitative interviews with children, parents and health professionals to identify how OI impacts on the quality of life and well being of children and their family. This is the first stage in the development of a disease specific QoL measure for children with OI and aims to identify the concepts and dimensions of QoL that are important to children with OI and their family.

## Method

**Figure 1:**
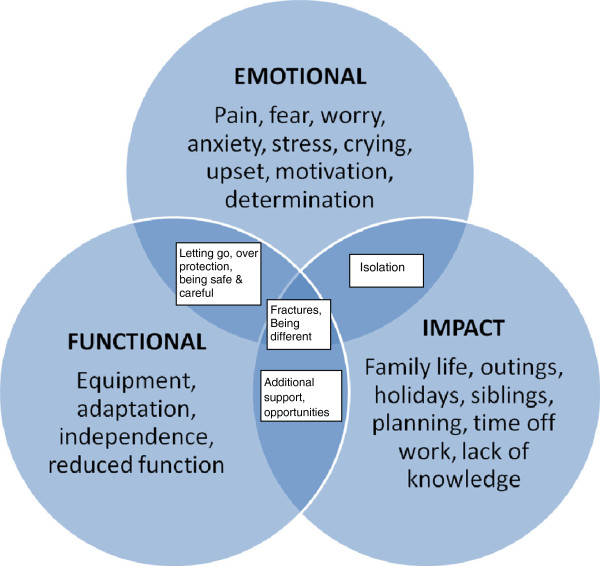
Hypothesized working conceptual framework, based on clinical expert opinion, for the health rated quality of life outcomes for a new patient reported outcome for children with OI.

The population was well known to the chief investigator and therefore allowed a hypothesized working conceptual framework, for the health related quality of life outcomes, to be developed (Figure 1) using clinical expert opinion. It was felt the main concept/theme would be the effect of fractures and that this theme may cross cut all others. The intended population was children with OI, aged 6–18, but the potential need for multiple age-related instruments was acknowledged. A frequency based Likert scale was deemed a suitable method for scoring and a three or five point Likert was considered.

Phenomenology was deemed the most appropriate methodology for this study. Phenomenology studies conscious experience as experienced from the first person point of view, and considers how individuals make sense of the world around them [24]. Through observation, attempts are made to gain access to an individual’s common sense thinking, how they interpret the reality within which they live.

Ten children with a diagnosis of OI were purposively sampled from those attending a tertiary Metabolic Bone Disease Clinic in the UK. Participants were approached gradually throughout the study, until saturation was achieved. Saturation of data occurs when no new information is heard or uncovered from the ongoing interviews. The development of QoL measures requires maximum variation within the sample and included children aged 7 to 18 with a range of severity and types of OI. Ten parents of children diagnosed with OI attending the same clinic, but independent of the children sampled; and five health professionals, located within the above mentioned clinic, specialising in the treatment of children with OI were also interviewed. (Table 1 describes the characteristics of the sample). Saturation was monitored continuously throughout recruitment. For completeness we choose to fully recruit to all participant groups to reduce the chance of missed themes.

**Table 1:** Characteristics of the samples

**Participant**	**Length of interview (minutes)**	**Age**	**Gender**	**Severity of OI (child)**	**Profession (Health Professional)**
C1 (child)	28	16	M	Moderate	
C2	26	16	F	Mild	
C3	13	8	F	Moderate	
C4	44	12	F	Moderate	
C5	34	6	M	Severe	
C6	28	13	M	Moderate	
C7	34	14	M	Mild	
C8	30	10	F	Mild	
C9	26	14	F	Severe	
C10	34	17	M	Severe	
P1 (parent)	52	30	F	Moderate	
P2	38	28	F	Moderate	
P3	50	38	F	Moderate	
P4	40	37	M	Severe	
P5	44	30	F	Mild	
P6	37	40	F	Severe	
P7	42	39	F	Moderate	
P8	18	42	F	Mild	
P9	43	44	F	Mild	
P10	42	52	M	Severe	
HP1 (Health professional)	48	44	F		Occupational Therapist
HP2	20	54	F		Nurse
HP3	34	28	F		Occupational Therapist
HP4	37	33	F		Physiotherapist
HP5	30	52	M		Consultant Medic

The local research ethics committee reviewed and approved the study protocol. A letter of invite was sent to participants requesting them to consent to being approached to take part in the study. The invitation included an information leaflet explaining the nature of the interviews, who would be present and what to expect, making it clear that participants could stop the interview at any time. Consent was gained from participating parents and health professionals. For children who agreed to participate, consent was gained from both parents and young adults (16–18 years) and assent was gained from children (7–15 years).

The interviewer [CH] was previously known to the children in a clinical role. This potential conflict of interest was acknowledged prior to ethics committee review, literature and opinions were sought, and following discussion the decision to use a clinician, known to the children, as the primary researcher was made. Children were given the choice to be interviewed alone or with a parent or carer present. Seven children were interviewed without their parent/carer, three chose to have their parent/carer present; these were the three youngest participants. The semi-structured interviews took place in a quiet room, away from the clinic or ward environment. All participants were made aware of the dictaphone recording the interview. Participants were informed that there were no right or wrong answers and that their views and opinions were what was required. An interview schedule was used to encourage ongoing focus throughout the interview. As you would expect of qualitative methodology several warm up questions were used at the onset; parental and child interviews included a warm up question asking them to tell me a little about themselves, health professionals were asked to describe their experience of working with the paediatric OI population.

Following this starter question, they were asked if having OI, or having a child with OI, had an effect on any of their daily activities. Parents and children were asked to discuss their daily routine, we hoped this would allow them to break down their daily activities and explore how OI impacted on their QoL. When interviewing the children probing was used to draw out any differences between themselves and their siblings or peers. Parents who had more than one child were asked if they felt they treated both children similarly.

The interviews were transcribed verbatim, and rechecked on several occasions for accuracy. A sample of interview transcripts (n = 5) were reviewed by a second qualitative researcher [WB]. The interview data was read, and re-read and reoccurring themes were identified. Significant themes and subthemes identified, extracted and organised, utilising framework analysis. Framework analysis is a systematic, visual method of analysing qualitative data and has five stages; familiarisation, identifying a thematic framework, indexing, charting, mapping and interpretation. It is inductive in nature allowing both a priori and emergent concepts to be combined. [25,26]. Particular attention was paid to identifying differing views in the data. Themes were then explored and any connections or overlap between themes considered. Anonymous data were shared and discussed at research meetings to establish consensus.

## Results

The first 25 individuals (10 children, 10 parents and 5 health care professionals), approached agreed to participate in the study. The age range of the children interviewed was 6 to 17 years; the parents interviewed had children with a mixed range of severity of OI. The health professionals included two occupational therapists, one physiotherapist, one specialist nurse and one consultant. All specialised in the treatment of children with OI with a wealth of experience, ranging from 2–16 years. Interviews ranged from 13 minutes to 52 minutes in length (See Table 1).

Most of the children appeared to feel at ease discussing at length their daily routine, identifying times and situations where they needed additional support, or had to do things differently from their siblings or peers. Although one child was able to talk about her Osteogenesis Imperfecta (OI) and describe her daily routine, she became upset when the interview touched on differences between her and her peers so the interview was stopped to avoid causing undue stress.

Health professionals due to their lack of constant proximity to the children and their families often discussed topics from both the child and the parent’s perspective. Children obviously described topics which were more relevant to them alone; parents discussed more family based topics, often with emphasis around the additional planning and organisation involved in caring for a child with OI.

Six main themes were identified as relevant to QoL from the analysis. These themes included; being safe, reduced function, pain, fear, isolation and independence. (See Table 2 for details). Although the complexity of the language used varied, all participant groups discussed the impact of the six themes on the QoL of children with OI, but the emphasis/spread was different for each group. Themes such as; reduced function (fractures and equipment) and isolation (being different) saturated quite early (n = 5), within the children interviewed. Being safe was discussed readily by all children interviewed, but more frequently by parents. The themes fear and being safe saturated earlier within the parent group, and fear was more readily discussed by health professionals. On reviewing the interview transcripts all the main themes were identified within the first eight interviews (4 children, 2 parents, 2 AHPs), although not all the sub themes were identified at this stage.

**Table 2:** Main themes and sub themes

**Main themes**	**Sub themes**
Being safe and careful	Avoidance of activities
	Trying to be safe
Reduced function	Reduced function with fractures
	Equipment/adaptation
	Tiredness/fatigue
Pain	General aches and pains
	Pain of fractures
	Pain relief
Fear	Fear of fracture
	Activities/handling
	Needle phobia
Isolation	Isolation from peers
	Being different
Independence	Pushing for independence
	Over protection
	Opportunities

### Being safe

There was no difference between children of primary or secondary age in their identification of the need to be safe. Younger children observed adults not letting them do certain activities to keep them safe and noticed how their parents would stay close to ensure safety. Secondary school aged children talked about having additional adult support to promote safety, but also how they avoided activities and busy areas to keep safe. Another more able bodied child did not need additional support to move around school, but stated “I leave at the same times as everyone else, I’m just extra careful”. Careful was a description used more often by the older children. (See Table 3 for quotations).

**Table 3:** Quotations to support themes

**Theme**	**Quotation**
Being safe	“Erm, there is an LSA that takes me into lessons, erm so that sort of if people were running and I sort of got knocked or something, there would be someone there.” Child (C7).
	“Yeah and what I would often see is she would do they same, but she would be right on the periphery.” Parent 9 (P9).
	“He wouldn’t do anything that would put him at risk, you know, he is not one for climbing trees, or sitting on someone’s shoulders or anything like that, so he is quite sensible (laughs).” P8.
	“They are very protected, and they are very cautious about who they allow near them, and so their social experiences are quite limited in those early days and the handling and the bonding is reduced.” Health Professional 1 (HP1).
Reduced function	“It’s, sometimes it can restrict me, but…I don’t know, it’s, I’m not the same if that makes sense....Yeah, because you are in a wheelchair like, sometimes it’s like harder to do things that other people could do with ease.” (P9).
	“He doesn’t do any after school activities, because by 3 o clock he’s wacked, he is whacked. He comes in erm, and he can like lay on the sofa for half an hour, an hour and just not do anything. (P3).
	“A lot of them don’t want to be reliant on someone to help them and they want to be able to manage by themselves but yet they have to accept, you know, help and just practical tasks like toileting and bathing and you know, getting in and out of bed of a morning, if you have a fracture, becomes that bit more difficult and time consuming.” (HP3).
Pain	“When I have a fracture, it is obviously very painful, but what upsets us the most is the fact of the consequences, because I mean I have had that many I believe that I am used to the pain and in comparison the screaming and the crying as I grew up, now I don’t really cry, I just you know, emphasise that I am in pain, but the worse thing is the consequences. (C10).
	“It’s just really hard sometimes and for me when I see her in pain I feel quite guilty about that, because I know that it’s obviously come from me. (P2).
	“Earlier pain management and almost the parent is the only person really who is there early enough, because you never get the same doctor twice and you never get the same treatment twice, and I think there needs to be some sort of care plan in place where the parents know that they are allowed to give..” (HP4).
Fear	“More scared of breaking a bone, I was always, it would hold me back a lot from doing more activities.” (C1).
	“I’d love to take the kids there, but I couldn’t, I’d, I’d, I’d just be too scared, I’d be just, there’d be too many people who were going in their own directions and I would be too frightened to do that.” (P7).
	“When they first go to school, schools are terrified of them whether they are mildly affected or severely affect, it doesn’t really matter, they are all terrified. And the child will be excited about going to school, and everybody is missing that.” (HP1).
Independence	“I’ve always been independent, because I’d prefer to be independent. They have said I can have a scribe for my GSCEs, but I don’t want one, I’d rather write it myself.” (C2)
	“Because they are very independent now they have been on pamidronate, well, no, Erin especially, she doesn’t rely on wheelchairs very much at all now, so she’s her own independent 12 year old.” (P7)
	“When it comes to independence at home…and there is this sort of letting go process, and they learn to drive, and have their independence that way and it’s a very exciting time for them, but it is very nerve racking for families to let them go.” (HP1)
Isolation/being different	“It makes me feels a bit left out because I can’t do a lot of things that I want to do.” (C8).
	“The birthday party invite would come, it’s a skating party, it’s a roller blade-ing party. It’s a horse riding party, no we can’t go.” (P9).
	“They are not going to be able to engage in the same range of activities. They may be limited sometimes in terms of what they do, not simply in terms of their physical disability, but by other people’s perceptions of what that disability means.” (HP5)

Parents, like the older children, talked about keeping their child safe and avoiding fast and unpredictable activities. Parents also observed their child remaining on the periphery of activities and not taking risks. Whereas health professionals observed parents from an early stage doing everything they could to keep their child safe from fracture.

### Reduced function

Primary school children reported using wheelchairs because they became tired if they had to walk a long distance, and that their function was reduced when on crutches if they had to open doors or carry something. One young child described going in his wheelchair “Because my legs got tired”. Older children talked about having to do things in a different way. They did not always talk about missing out on activities, but had to adapt the activities or choose to do a slightly different activity alongside their more able bodied peers. This was especially apparent at times when a fracture involved a dominant hand.

Others described the different equipment they used to improve their function, these included walking with crutches and using wheelchairs for mobility. One teenager required a science stool with a backrest, and commented that her school had to order a special one. Another reported using “chunky pens, so that I can grip them easier”.

Parents and health professionals also observed reduced function, but commented more on the additional help children with OI required, particularly if they had sustained a lower limb fracture and were none weight bearing. They described the tiredness they observed in their children when they experienced a busy school day. Another health professional described the restrictions placed on some activities for older children such as; trampolining, horse riding, high impact sports and PE. They felt younger children were also restricted from simple activities like slides at a play ground, which if pursued could lead to fractures, demonstrating a link to being safe.

### Pain

Younger children talked about pain in terms of “ouchys” and things “hurting”. Older children described pain, hurt and ache, often relating to fractures, but occasionally just the general aches and pains experienced by people with OI. One older child described his back ache as “always there, it comes and goes like a pain threshold”.

Parents talked about finding it hard to see their child in pain, and those who had OI themselves felt guilty for passing on the gene, when the pain experienced following a fracture was discussed. Some parents commented on how much pain their child had suffered before they had received bisphosphonates. They talked about their child appearing lifeless and finding activities difficult due to their level of pain. Some parents discussed the advantages of early pain relief and splinting immediately following fracture and this was mirrored in the comments made health professionals.

### Fear

Younger children had no concept of fear, whereas secondary school aged children described how fear of fractures would hold them back from undertaking some activities, they were fearful of busy or dangerous areas and some reported needle phobia.

Parents did describe fear in their children of all ages; they observed fear of some activities and needle phobia, but also reported their extended families fear of handling their child, and the fear they experienced when their child went to school. One parent described her brother as “panicking he might break her”, when she had asked him to hold her daughter, and noted that most of her family chose not to pick up and handle her. Health professionals observed fear in parents, children and other professionals with regard to handling and fractures; they did not differentiate for age where fear was concerned. They noticed children with OI often limited the number of people in their family that were allowed to handle them and pick them up. One health professional felt that older children often became anxious when they handled them for the first time, particularly if they had only been handled by their parents.

### Independence

Parents and children differed substantially in their discussions around independence. Secondary school children described striving for independence, and preferring to be independent even when they had sustained a fracture. One child described having pushed to be like everyone else at school, only agreeing to sit out of PE when their legs became too tired to carry on. Others talked about walking or propelling themselves to school with their friends. Younger children did not talk of independence at all; it is assumed this was due to age and expectation. Parents on the other hand described struggling from an early age with letting go and over protection. They observed their child’s drive and motivation for independence, but often reported their need to attend extracurricular activities alongside their child, and in some cases acknowledged their inability to let go.

The health professionals observed several times within the child’s life when they struggled for independence against the overprotection of their families and school. They also commented on the difficulties surrounding children moving into education and no longer within the sole care of their parents. All health professionals described the young OI population as motivated and determined, striving for independence. One stated “they are a great example of what can be achieved by determination and courage”.

### Isolation/being different

The final theme described by children, parents and health professionals was isolation or feelings of being different. Younger children talked about not being allowed to play football or run fast. They described feeling left out of some outdoor school trips, not being invited to parties, and feeling sad that there were things they could not do. One child became upset when she talked about being unable to skip or run and the interview was terminated as a result. The older children echoed the thoughts of the younger ones; they felt left out of some activities which were potentially dangerous to them because of their OI and found some extracurricular activities became too physically demanding as their peers became older and stronger.

Parents and health professionals observed this isolation in the children they cared for. They reported children being left out of PE and sports day, but also not being able to keep up with their peers or siblings. In some instances parents commented on their child looking different because of the equipment they required, and the effect this had on a child who wanted to look like everyone else. One health professional felt that children with OI could access most opportunities with some modification, but went on to say this took “extra effort from teachers and schools, social groups and medical professionals.”

## Discussion

This study used qualitative interviews with children, parents and health professionals to identify how OI impacts on the quality of life and well-being of children and their family and what issues are important. It was the first stage in the development of a disease specific quality of life measure for children with OI. From the 25 interviews undertaken with children, parents and health professionals, six main themes were identified, extracted and organised using framework analysis and included; being safe and careful, reduced function, pain, fear, isolation and independence.

There is a paucity of research on the views of both children and parents on the impact of OI on QoL.

A recent Portuguese descriptive case study [27] described some similar themes to those identified here. They interviewed children with OI, their siblings and parents. Reporting themes such as; consequences of fracture and impairment, weakness and vulnerability, exclusion at school, worries surrounding pain and susceptibility to fracture and positivity.

Stevens [18] interviewed 74 children aged 7–11, from two city schools, about their quality of life. Her main aim was to develop a health utilities index for children, and used the interviews as a means to identify themes. The majority of children were in good health (93%), no individuals reported complex disability. Her themes included; worried, sad, annoyed, hurt, learning, daily routine, tired, joining in activities, sleep, embarrassed and jealous.

Quality of life is a difficult concept to describe, and descriptions vary across the literature. Previous definitions [14] are based around functional ability and health status, but both experience and the literature tells us that quality of life is not necessarily based on a child’s ability to function, and there is no evidence to suggest this is the case [28]. Difficulties arise when attempting to measure quality of life, if it is not well predefined. Some define it as functional ability or a sense of wellbeing [19], others report health related QoL [29]. It is hard to make comparisons between research papers, if different definitions are used. From the number of themes identified within the interviews, it can be seen that only a small proportion relate to functional ability. Reduced function was more often mentioned if the child had sustained a fracture and this was true for all ages and interviewees.

Several studies have discussed the difference found between children and their parents with regard to their understanding of QoL [15,16,30,31]. This research supports the view that differences also exist between the children and their health professionals [32]. It also highlights a large number of similarities between children and their parents within the six main themes identified, although there were several important disparities apparent within the interviews. Parents talked about how their child’s OI affected their own QoL; their ability to work; undertake family activities; and the additional planning required to achieve some outdoor pursuits. Parents on the whole, failed to mention their child’s independence, without discussing the over protection they felt it necessary to provide. Eiser [16] stated that we shouldn’t expect strong correlations between parent and child ratings, but it is important to identify those contexts in which parents can be expected to make an accurate judgement. Parents are not therefore, necessarily considered an accurate source of information when identifying issues around their child’s QoL [15,30,33]. For this reason parents may well be a suitable advocate when reporting on themes such as; reduced function, being safe and careful, and pain, they may not be an adequate proxy overall.

The research undertaken found strong agreement between the groups for themes; being safe and careful, functional ability particularly following fracture, isolation from activity and pain. Although all three groups agreed on the nature of these themes, their justification for the theme was different. There was not agreement however when discussing independence and fear. Young children of all abilities are cared for by their families, so striving for independence is often not anticipated in this age group. With the exception of the very young children, fear was a theme reported by all those interviewed. The impact of fear varied across the group; children had a fear of certain activities that previously resulted in a fracture, parents fear related to anxiety of handling and of safety/separation when the child was at school/nursery or play dates. As expected younger children were unaware of the over protective actions of their parents which is a stark contrast to the older children expressing their dislike of parents attempts to keep them safe and doing everything for them.

As anticipated many of the themes have a link to fractures; the fear surrounding potential fracture, the avoidance of sustaining a fracture, the resultant pain and reduced function following fracture, and the effect of repeating an activity which previously lead to a fracture. No other literature or QoL measure contains any link to fractures and the effect they can have on a child’s QoL. Several of the themes identified from the interviews are similar to those in other QoL measures. Independence, function and pain are included within the CHIP-AE [34], TACQOL [35], and DISABKIDS-37 [36], but this lack of connection to fractures demonstrates the need for an OI specific QoL measure.

## Conclusion

This data presents the first step in developing items for a disease specific QoL measure for children with OI. Six main themes were identified; being safe and careful, reduced function, pain, fear, isolation and independence. There was generally good agreement between the three groups of interviewees, although discrepancies did occur between parents and children, with regard to the themes independence and fear. Several of the themes uncovered showed similarity to other QoL measures, but the addition of being safe and careful, particularly in relation to fractures, demonstrated the need for a disease specific measure for children with OI. Further research in the form of focus groups will take place to validate these themes. The themes identified and vocabulary used by the children will directly inform development of the items within the OI specific QoL measure.

## Abbreviations

OI: Osteogenesis imperfecta; QOL: Quality of life; HUI III: Health utilities index III; PEDI: Pediatric evaluation of disability inventory; SPPC: Self-perception profile for children; AHP: Allied health professional; HP: Health professional; GP: General practitioner.

## Competing interests

The author(s) declare that they have no competing interests. Conflict of interest is discussed within the paper in relation to CH and their role as a paediatric physiotherapist known to the participants.

## Authors’ contribution

CH carried out the 25 semi structured interviews, undertook the transcription, framework analysis, interpretation, theme generation and drafted the manuscript. SJW and WB supervised the conduct of the research. WB supported the data analysis, reviewing transcripts, checking the accuracy of interpretation and supported theme generation. All authors contributed to the production of the manuscript. All authors read and approved the final manuscript.

## Authors’ information

CH (MSc, PGCE, MCSP) is a practicing paediatric physiotherapist within a specialist Metabolic Bone Disease Service and undertook this research as part of a PhD qualification.
